# Comparison of the Long-Term Outcomes of Limb Salvage vs. Amputation in Lower Limb Vascular Trauma Patients: A 10-Year Follow-Up Study

**DOI:** 10.7759/cureus.95505

**Published:** 2025-10-27

**Authors:** Sadegh Abaei, Samin Shokravi, Niki Tadayon

**Affiliations:** 1 General Surgery, Shahid Beheshti University of Medical Sciences (SBMU), Tehran, IRN; 2 Vascular Surgery, Shahid Beheshti University of Medical Sciences (SBMU), Tehran, IRN; 3 Vascular Surgery, Shohada Tajrish-Shahid Beheshti University of Medical Sciences (SBMU), Tehran, IRN

**Keywords:** amputation, heath-related quality of life, lower limb, revascularization, trauma

## Abstract

Background

Vascular revascularization of ischemic tissues is critical to prevent neuromuscular damage and preserve limb function. This study compared outcomes in lower limb trauma patients undergoing revascularization or amputation over a 10-year period.

Methods

We conducted a prospective observational study of 88 patients treated at a tertiary referring center. Demographic and injury data were collected, and functional outcomes have been allocated based on clinical criteria and surgeon judgment. Treatment outcomes included mortality, wound infection, rehabilitation requirements, and return to work.

Results

The amputation group was significantly older and experienced more surgical site infections than the revascularization group, whereas mortality and most functional outcomes did not differ significantly. Revascularization offered comparable long-term functional and psychosocial outcomes with fewer complications.

Conclusions

These findings suggest that when feasible, limb revascularization should be prioritized to optimize recovery and quality of life, as it yields similar survival and function with fewer complications than amputation.

## Introduction

Lower limb-threatening injuries are associated with high mortality, severe disability, and loss of limb function [1-4]. Vascular injuries represent about 2% of trauma admissions and require prompt revascularization to prevent ischemia [5]. Advances in diagnostics, endovascular techniques, and critical care have improved limb salvage, but selecting between reconstruction and amputation remains complex [6-8]. Predictive tools like the Mangled Extremity Severity Score offer limited guidance [9-11]. In low-resource settings, restricted access to level I trauma care further complicates management. There is ongoing debate over whether primary amputation yields better long-term function, pain control, and quality of life than attempted revascularization. Previous studies often have small sample sizes and short follow-up periods. To address these gaps, this prospective 10-year study compares long-term functional outcomes among patients undergoing lower-extremity revascularization or amputation after traumatic arterial injury, evaluating demographic, clinical, and socioeconomic factors that may influence recovery [12-15].

## Materials and methods

Study population

The study population included patients older than 16 who suffered high-energy trauma below the distal femur and were admitted to the level I trauma department. High-energy trauma was defined as major soft-tissue injuries (degloving or severe crush or avulsion injury), dysvascular limbs (knee dislocations, closed fractures of the tibia, or penetrating wounds with vascular injury), and severe foot and ankle injuries (Gustilo grade IIIB ankle fractures, all grade III intra-articular fractures of the distal tibia (pilon), and severe crush or avulsion injury). The following groups of people were not included in the study: those with a Glasgow Coma Scale score of less than 15, indicating some impairment in consciousness 21 days after hospitalization or discharge; those with a spinal cord deficit, prior amputation, or third-degree burns; those who were transferred more than 24 hours after the injury; those who did not speak Persian; those with a documented psychiatric disorder; and those who have had surgery within the last year.

The current study's cohort consists of patients who have suffered traumatic injuries to their lower limbs and have undergone limb amputation surgery or vascular reconstruction at Shohadaye-Tajrish Center since 2012. Between March 2012 and June 2022, we enrolled 104 patients. Eleven patients with bilateral injuries who met the study criteria were excluded from the current analysis. An additional 15 patients were excluded due to a lack of follow-up. In total, 44 patients were in the amputation intervention group and 44 in the vascular reconstruction treatment group.

Procedures

The physical and psychosocial subscores of the Sickness Impact Profile (SIP) were used to assess functional status [16-18]. The SIP is a multidimensional measure of self-reported health status that includes 136 statements about limitations in 12 functional categories: (1) walking, (2) mobility, (3) body care and movement, (4) social interaction, (5) alertness, (6) emotional behavior, (7) communication, (8) sleep and rest, (9) eating, (10) work, (11) home management, and (12) recreation. Respondents are asked to support statements that describe their current health status. The whole instrument is scored; an investigator administers the entire SIP instrument in person. A SIP score of more than 10 indicates significant disability, while variances of 2 to 3 points indicate notable changes in function. Overall SIP scores range between 2 and 3 points for the general population [19-21].

The trauma surgeon assessed patients and recommended limb treatment based on clinical judgment and patient preferences. Comparisons of outcomes based on the SIP were corrected for confounding factors, such as the patients' characteristics and injuries. This was necessary because treatment assignments were not randomly assigned. The injuries were classified prospectively based on the type and extent of bone damage, the extent of soft-tissue injury, and the initial pulse evaluation and plantar sensation. The level and type of closure (standard, elective skin flap, and best-possible skin coverage, including split-thickness skin grafts and free-tissue transfers) were also considered. Shock was defined as having a systolic blood pressure of less than 90 mm Hg before resuscitation. To account for complications' impact on recovery, a variable was created to indicate rehospitalization for one or more conditions: late amputation or stump revision, fracture non-union, hardware failure, flap failure, wound infection, or osteomyelitis.

It was crucial to fully characterize the patients and injuries in the two treatment groups and adjust for differences due to the non-random nature of the treatment assignment in this study. The factors hypothesized to influence the type of treatment (amputation or reconstruction) and the SIP outcomes were the characteristics of the study injury and injuries sustained in conjunction with the study injury, post-acute care complications, and patient and environmental factors.

Statistical analysis 

Data analysis was performed in SPSS version 21 (SPSS Inc., Chicago, IL, USA). P-value ≤ 0.05 was considered statistically significant. Mean and standard deviation were used to express quantitative data, and numbers and percentages were used to express qualitative data. The normality assumption was checked using the Kolmogorov-Smirnov test, and the relationship between the disease and qualitative data was studied using Chi square.

Because many variables described the nature and extent of injuries, a summary score showed how likely a patient would need an amputation based on their injury profile. This score was created with the help of logistic regression, which modeled the decision to amputate or reconstruct based on the characteristics of the injury. Traumatic amputations were assigned a summary score of 1 because the underlying injury features were not documented. Multivariate models that included all aspects of the injury as independent factors gave results equal to those obtained using models with summary scores; hence, the more economical model, which employed the summary score, is provided.

Ethical considerations

Patients' participation in this study is contingent on their informed consent, and all patients can withdraw at any stage. In this study, there was no intervention by the researcher for the patients. All patients were adequately informed about the plan and entered the study after providing informed consent to engage in the research plan.

If the patients refused to participate in this trial, their treatment process and benefit from the treatment services remained the same. All patients' information was kept private, and confidentiality was maintained without their names. If necessary, data were coded to keep patient confidentiality.

## Results

The amputation group was significantly older and experienced more surgical site infections than the revascularization group, whereas mortality and most functional outcomes did not differ significantly. Revascularization offered comparable long-term functional and psychosocial outcomes with fewer complications (Table 1).

**Table 1:** Baseline characteristics of the patients.* *Because of rounding, not all percentages sum to 100. ^†^The income level was estimated according to the method of Fisher. ^‡^Scores for the injury severity score can range from 1 to 75, with scores of 17 or higher indicating severe injury.

Characteristic	Reconstruction (N = 88)	Amputation (N = 88)	Characteristic	Reconstruction (N = 88)	Amputation (N = 88)
			Overall injury		
Age			Injury severity score (%)^‡^		
>40 yr (%)	31.5	36.7	<13	63.2	66.7
Mean (%)	32.4	35.2	13-17	19.3	11.8
Male sex (%)	75.0	80.8	>17	14.4	23.2
Education (%)			Ipsilateral leg injury (%)	24.2	29.7
Some high school	28.4	31.0	Contralateral leg injury (%)	20.4	15.6
High school graduate	40.4	40.4	Shock (%)	20.7	29.9
Some college	31.3	28.6	Tibia fracture (%)		
Smoking history (%)			None	54.2	57.3
Never smoked	35.4	31.0	Simple (AO type A)	9.9	1.2
Former smoker	27.6	31.3	Wedge (AO type B)	38.8	23.2
Current smoker	36.4	37.2	Complex (AO type C)	3.6	25.3
Income as a percentage of poverty level (%)^†^			Open foot facture (%)	12.4	21.2
<125%	20.2	23.2	Pulse absent at initial evaluation (%)	11.3	54.2
125%-200%	34.9	35.6	Plantar sensation absent (%)	6.2	56.2
>200%	46.7	48.2			
Health insurance (%)					
None or public	48.5	46.2			
Private	22.1	19.6			

Furthermore, 24 people (27.3%) used cigarettes, eight people (9.1%) used illicit drugs, and seven people (8%) used alcohol. The study's findings show that the two groups were similar regarding cigarette, drug, and alcohol usage, with no discernible difference between them (Table 2).

**Table 2:** Comparison of habitual history between the two groups.

Variable		Total population	Amputation group	Revascularization group	P-value
		Number (%)			
Cigarettes	Yes	24 (27.3)	12 (27.3)	12 (27.3)	P = 1.00
	No	64 (72.7)	32 (72.7)	32 (72.7)	
Illicit drugs	Yes	8 (9.1)	4 (9.1)	4 (9.1)	P = 1.00
	No	80 (90.9)	40 (90.9)	40 (90.9)	
Alcohol	Yes	7 (8)	4 (9.1)	3 (6.8)	P = 0.998
	No	81 (92)	40 (90.9)	41 (93.2)	

**Figure 1:**
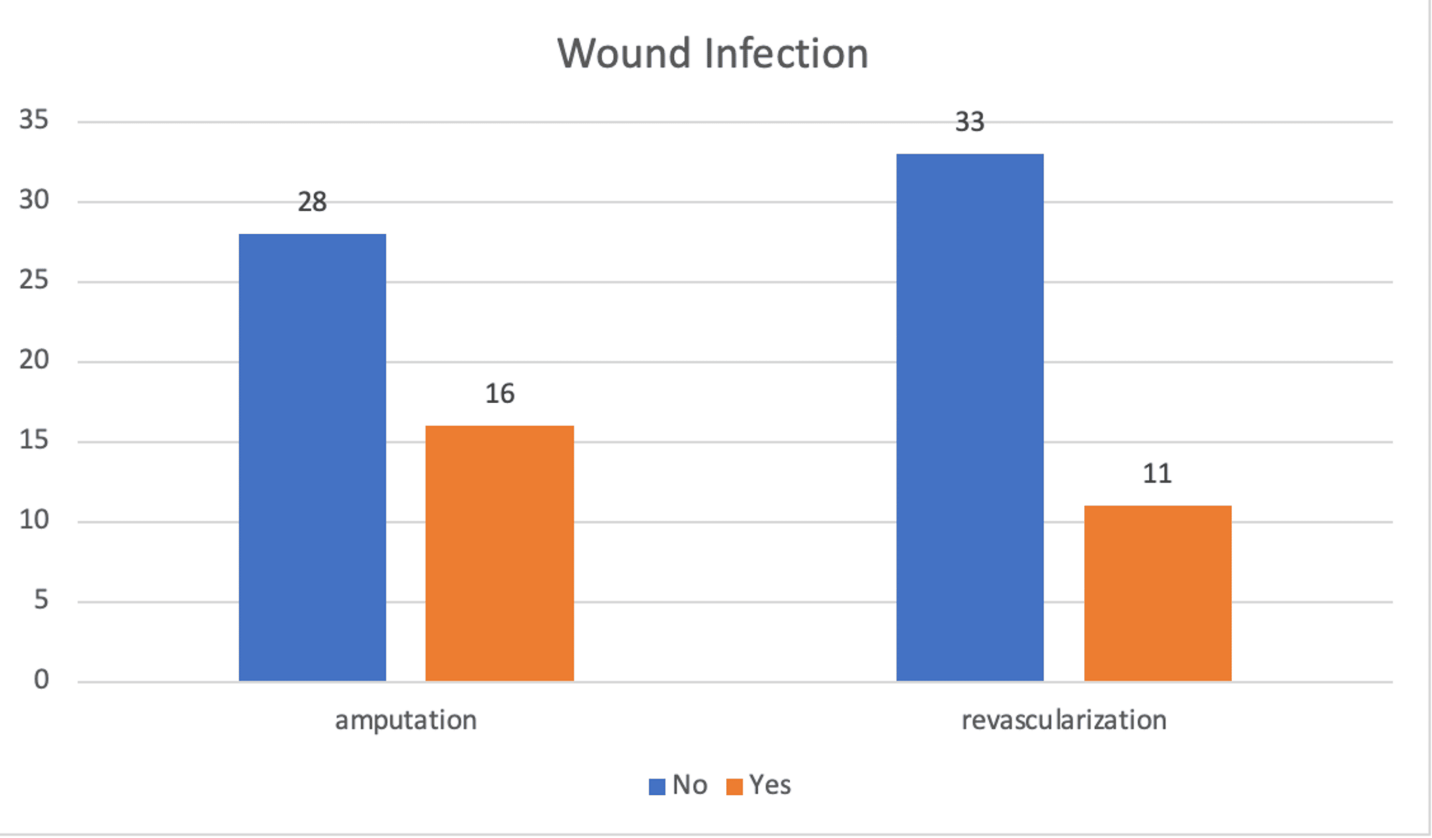
Comparison of wound infection in two treatment groups.

In terms of observed outcomes, 27 persons were infected. According to the findings, the amputation group had more infections than the vascular reconstruction group. However, no statistically significant difference was found between the two groups (P = 0.248) (Figure 1).

**Figure 2:**
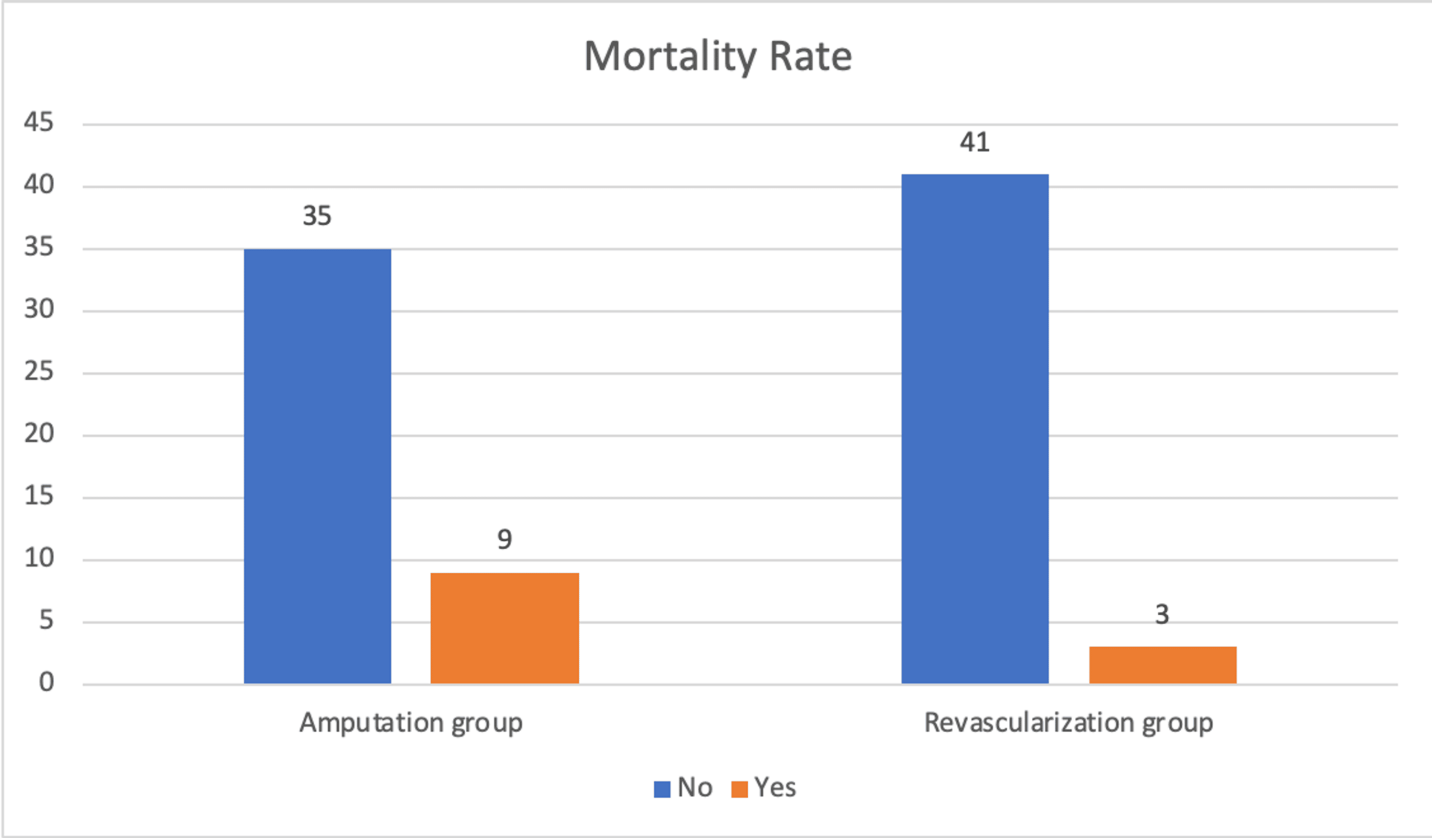
Comparison of mortality rate in two treatment groups.

In addition, 12 participants overall (three in the vascular reconstruction group and nine in the amputation group) did not make it through the study. The mortality rate was greater in the group that underwent amputation (20.5% versus 6.8%). However, this sum was not statistically significant (P = 0.06) (Figure 2).

The postoperative outcomes of the two groups were compared. After surgery, 31 patients (75.6%) required rehabilitation for their lower extremities. No statistically significant difference between the two groups was seen in the need for limb-specific physical therapy following surgery (P = 0.377) (Table 3). Additionally, psychiatric therapy was required for 23.8% of the study population, and the results showed no statistically significant difference between the two treatment groups (P = 0.890). Compared to before the operation, most participants (about 65%) reported their general condition as satisfactory or outstanding. Only seven patients (9.2%) said they were in a more severe condition after surgery than earlier. The findings showed no significant differences when comparing the two groups' conditions before and after the operation (P = 0.693) (Table 3).

**Table 3:** Comparison of the need for limb-specific physical therapy between the two groups.

Variable		Total population	Amputation group	Revascularization group	P-value
		Number (%)			
Physiotherapy is needed	Yes	23 (28.7)	13 (33.3)	10 (24.4)	P = 0.377
	No	31 (75.6)	26 (66.7)	31 (75.6)	
Psychiatric consultation is needed	Yes	61 (76.3)	30 (76.9)	31 (75.6)	P = 0.890
	No	19 (23.8)	9 (23.1)	10 (24.4)	
General condition compared to before the operation	Yes	7 (9.2)	3 (8.6)	4 (9.8)	P = 0.693
	Average	20 (26.3)	11 (31.4)	9 (22)	
	Good	37 (48.7)	17 (48.6)	20 (48.8)	
	Excellent	12 (15.8)	4 (11.4)	8 (19.5)	

**Figure 3:**
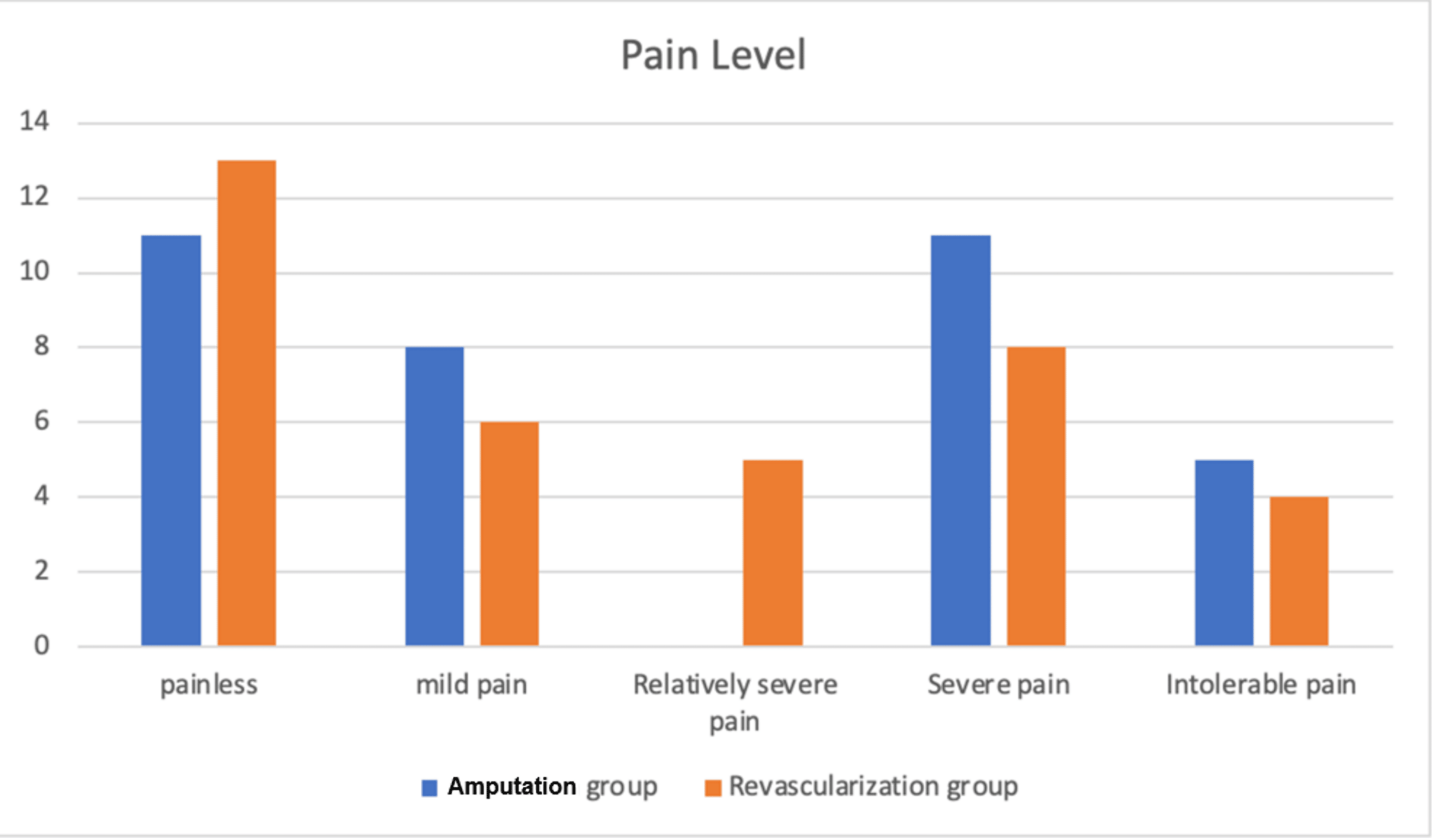
Differences in pain intensity between the two groups.

According to the findings of this study, the pain level in the amputation group is higher than that in the vascular reconstruction group. However, there was no statistically significant difference between the two groups (P = 0.197). The pain experienced by each treatment group is displayed in Figure 3.

After rehabilitation, the affected joints' range of motion (ROM) was compared between the two groups. The study found no statistically significant difference between the two groups regarding mobility restrictions (P = 0.767). In addition, there was no significant difference between the two study groups regarding normal daily activities (P = 0.735) (Table 4).

**Table 4:** Comparison of motor status in the two studied groups. ROM: range of motion

Variable		Total population	Amputation group	Revascularization group	P-value
		Number (%)			
ROM reduction	No	23 (30.3)	10 (28.6)	13 (31.7)	P = 0.767
	Yes	53 (69.7)	25 (71.4)	28 (68.3)	
Normal daily activities	No	5 (6.8)	2 (5.7)	3 (7.7)	P = 0.735
	Yes	69 (93.2)	33 (94.3)	36 (92.3)	
Participating in rigorous physical activity	No	10 (15.6)	6 (17.6)	4 (13.3)	P = 0.738
	Yes	54 (84.4)	28 (82.4)	26 (86.7)	

The study compared two groups of people based on their job activities. Out of all the participants, 55 people returned to work - 27 in the amputation group and 28 in the vascular reconstruction group. The results indicated no significant difference between the two groups in terms of returning to work (P = 0.568), as shown in Table 5.

**Table 5:** Comparison of job activities in the two studied groups.

Variable		Total population	Amputation group	Revascularization group	P-value
		Number (%)			
Return to work	No	17 (23.6)	7 (20.6)	10 (26.3)	P = 0.568
	Yes	55 (76.4)	27 (79.4)	28 (73.7)	
Decreased performance at work	No	30 (41.1)	13 (38.2)	17 (43.6)	P = 0.643
	Yes	43 (58.9)	21 (61.8)	22 (56.4)	
		Mean (standard deviation)			
Time to return to work (month)		10.62 (84.6)	9.6 (6.1)	11.61 (7.47)	P = 0.380

The study findings indicate that the amputation group had a faster return to work compared to the vascular reconstruction group. However, statistical analysis revealed no significant difference between the two groups, with a P-value of 0.380.

The study found that there was no significant difference in work performance between the two treatment groups. This was indicated by a P-value of 0.643 (Table 5).

Out of the total number of people observed, 39 individuals (51.3% of the sample) showed a decline in their social functioning. However, there was no noticeable difference between the two treatment groups in terms of their social impairment (as indicated by a P-value of 0.985 in Table 6).

**Table 6:** Social functioning.

Variable		Total population	Amputation group	Revascularization group	P-value
		Number (%)			
Social communication dysfunction	No	37 (48.7)	17 (48.6)	20 (48.8)	P = 0.568
	Yes	39 (51.3)	18 (51.4)	21 (51.2)	
Experience periods of anxiety	No	52 (68.4)	22 (62.9)	30 (73.2)	P = 0.335
	Yes	24 (31.6)	13 (37.1)	11 (26.8)	
Experience periods of depression	No	45 (59.2)	18 (51.4)	27 (65.9)	P = 0.202
	Yes	31 (40.8)	17 (48.6)	14 (34.1)	
Trouble sleeping	No	42 (55.3)	16 (45.7)	26 (63.4)	P = 0.122
	Yes	34 (44.7)	19 (54.3)	15 (36.6)	

## Discussion

Older age in the amputation group and higher infection rates contrasted with similar mortality and functional outcomes across treatments. These results mirror prior work emphasizing early revascularization and tailored postoperative care. Given the rarity and heterogeneity of vascular trauma, standard guidelines are elusive; emerging endovascular techniques may improve outcomes, yet availability is limited. Treatment should be individualized based on injury severity, patient preferences, and resources, and larger multicenter studies are needed to inform decision-making.

Limitations

The small sample size, observational design, and self-reported socioeconomic data limit generalizability and may introduce bias; not all complications were captured.

## Conclusions

Amputation produced higher infection rates and slower return to work, but overall functional and psychosocial outcomes were comparable. When feasible, revascularization should be favored. Larger, multicenter studies with predictive models are needed.
